# Supplementary material for: Flexible TiO2/PVDF/g-C3N4 Nanocomposite with Excellent Light Photocatalytic Performance
Source: Polymers (Basel). 2019 Dec 31;12(1):55. doi: 10.3390/polym12010055 (PMC7023571; doi:10.3390/polym12010055)
Supplement: Supplementary file 1 [file polymers-12-00055-s001.pdf]

Supporting Information

Flexible TiO_2/PVDF/g-C_3N_4 Nanocomposite with Excellent Light Photocatalytic Performance

Tong-Tong Zhou,[a)†] Feng-He Zhao,[b)†] Yu-Qian Cui,[c)] Li-Xiang Chen,[a)*] Jia-Shu Yan,[a)]

Xiao-Xiong Wang,[a)] and Yun-Ze Long [a),d)*]

Fig. S1 HRXPS of F 1s for TPCN2.

Fig. S2 The curves of $(h\nu*F(R))^{1/2}$ vs hν originated from the diffuse reflectance spectra in **Fig. 6**.

Fig. S3 Reaction rate constant for degradation RhB over different photocatalysts.

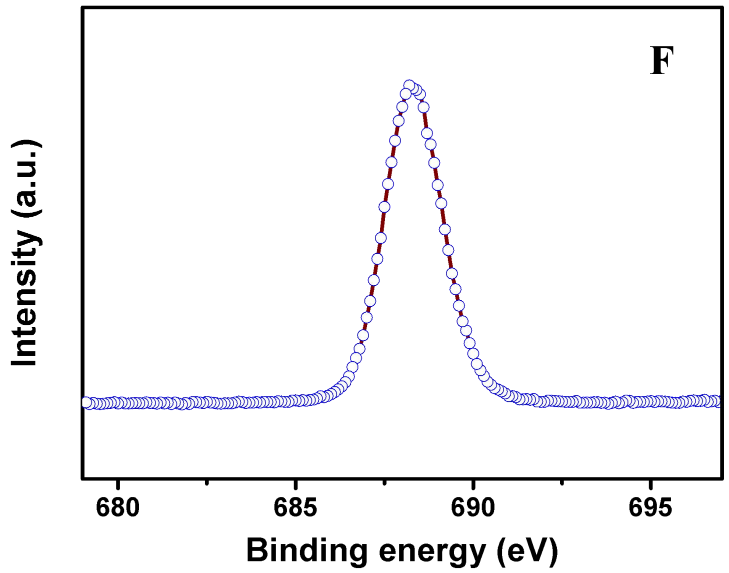

Fig. S1 HRXPS of F 1s for TPCN2.

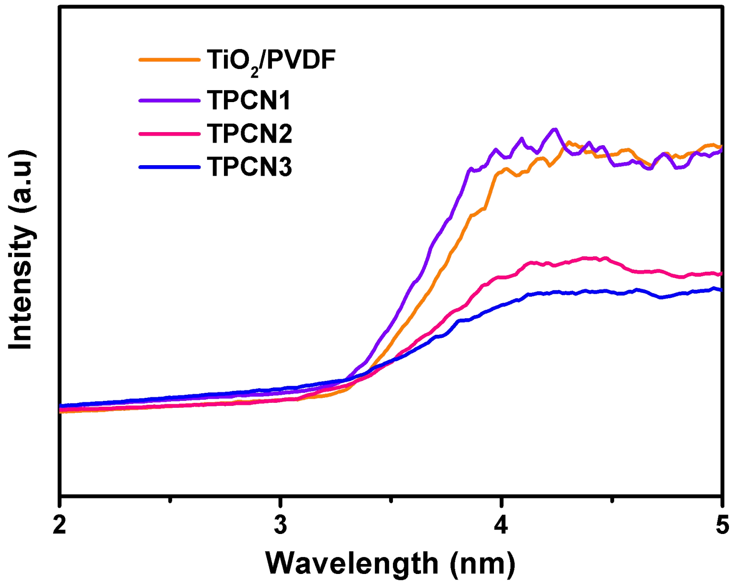

Fig. S2 The curves of $(h\nu*F(R))^{1/2}$ vs hν originated from the diffuse reflectance spectra in **Fig. 6**.

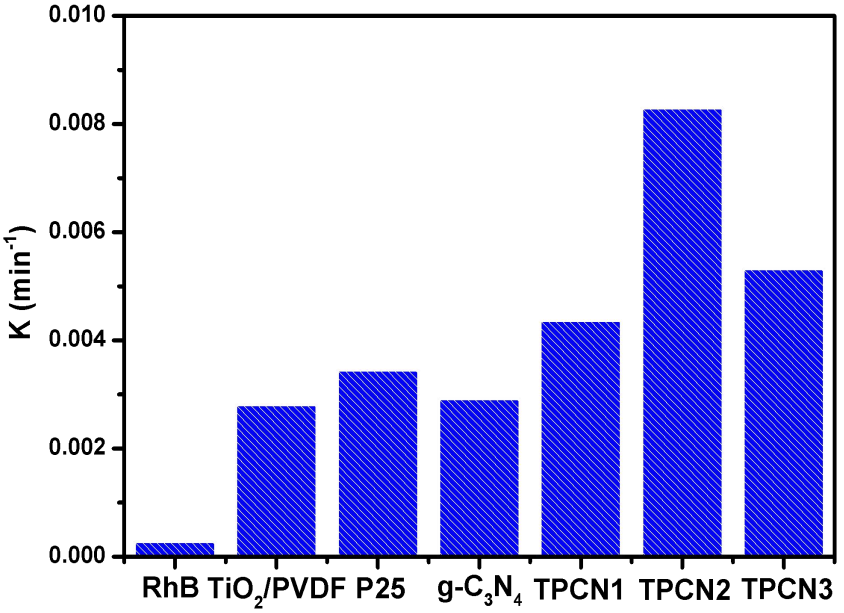

Fig. S3 Reaction rate constant for degradation RhB over different photocatalysts.
